# “Omics” Tools for Better Understanding the Plant–Endophyte Interactions

**DOI:** 10.3389/fpls.2016.00955

**Published:** 2016-06-29

**Authors:** Sanjana Kaul, Tanwi Sharma, Manoj K. Dhar

**Affiliations:** School of Biotechnology, University of JammuJammu, India

**Keywords:** endophytism, microbiome, endophytes, genomics, metagenomics

## Abstract

Endophytes, which mostly include bacteria, fungi and actinomycetes, are the endosymbionts that reside asymptomatically in plants for at least a part of their life cycle. They have emerged as a valuable source of novel metabolites, industrially important enzymes and as stress relievers of host plant, but still many aspects of endophytic biology are unknown. Functions of individual endophytes are the result of their continuous and complex interactions with the host plant as well as other members of the host microbiome. Understanding plant microbiomes as a system allows analysis and integration of these complex interactions. Modern genomic studies involving metaomics and comparative studies can prove to be helpful in unraveling the gray areas of endophytism. A deeper knowledge of the mechanism of host infestation and role of endophytes could be exploited to improve the agricultural management in terms of plant growth promotion, biocontrol and bioremediation. Genome sequencing, comparative genomics, microarray, next gen sequencing, metagenomics, metatranscriptomics are some of the techniques that are being used or can be used to unravel plant–endophyte relationship. The modern techniques and approaches need to be explored to study endophytes and their putative role in host plant ecology. This review highlights “omics” tools that can be explored for understanding the role of endophytes in the plant microbiome.

## Introduction

Endophytes are the microorganisms that reside within various tissues of the host plant in a commensal or beneficial manner. They can be considered as promising source of natural metabolites holding plethora of potential benefits in medical field (Strobel and Daisy, 2003; Strobel et al., 2004; Kaul et al., 2012; Premjanu and Jayanthy, 2012; Mousa and Raizada, 2013). A large number of compounds with significant bioactivities have been isolated from endophytes (Strobel et al., 2004; Kaul et al., 2012; Kusari et al., 2014). The scientific community has explored number of medicinal plants until now, for their endophytic repository. Inspite of a long list of reports on bioactive compounds from endophytes, commercial production of such compounds is still in its infancy (Kusari et al., 2014). Moreover, endophytes also have the ability to benefit the host plants with biotic and abiotic stress tolerance as well as improved nutrient acquisition and plant growth promotion (Johnson et al., 2004; Rodriguez et al., 2008). Such an ability can be exploited as a novel strategy to mitigate the repercussions of world climate change on agricultural crops and land. Therefore, in order to realize the potential of endophytes in pharmaceutical and agricultural industry, integrative understanding of all aspects of endophytism is essential.

Earlier, scientist community focussed on unraveling the endophytic diversity and their metabolite potential but now-a days there is more thrust on deep understanding of the host plant–endophyte niche. Suryanarayanan (2013), has highlighted some gray areas of endophytism which need to be addressed viz. endophyte-host plant interactions; interactions among different endophytes of the same host plant, relation of endophytes with non-endophytic groups of the plant microbiome, life strategies of endophytes with respect to their saprotrophic and pathogenic counterparts, etc., Modern methodologies discussed in the review would be helpful to fill these gaps and thus enhance our knowledge about endophytism (Suryanarayanan, 2013).

## Genome Sequence Analysis

The basic physiological aspect of the endophyte host interaction is poorly understood. Therefore, identification, isolation and characterisation of genes involved in such beneficial interactions is critically important for the effective manipulation of the mutualistic association between the two. Endophyte genome analysis has provided a new tool to closely view the endophytism and to reveal the requisite features to harbor plants as a habitat. It has revealed the genes, important for the endophytic life style, as found common in the endophyte genomes such as genes for- nitrogen fixation, phytohormone production (IAA, GA, etc.), mineral acquisition (Fe, P, etc.), stress tolerance, adhesion and other colonization related genes (Fouts et al., 2008; Firrincieli et al., 2015; Martinez-Garcia et al., 2015). These traits explain the role of microbes in nutrient cycling as well as their ability to colonize plant endosphere.

Whole genome analysis of endophytic microbes has revealed the genetic features that directly or indirectly influence the various bioactivities as well as colonizing preferences. It aids in the identification of particular genes involved in mechanism of antibiotic resistance, antibiotic production, plant growth promotion, endophytic secretory system, surface attachment and insertion elements, transport system and other related metabolic mechanisms. Such studies have provided greater understanding of the ecology and evolution of endophytes. The presence of genes encoding *N*-acyl homoserine lactone synthases and hydrolases, hyperadherence factors, fusaric acid resistance proteins, etc., highlight the biotechnological potential of the endophytic bacterium *Pantoea ananatis* (Megias et al., 2016). Endophytic members of the fungal order sebacinales have raised considerable interest due to their plant growth promotion and stress tolerance potential (Weiss et al., 2011^1^). *Pirifomospora indica* (order sebacinales) represents a model for the study of symbiosis interactions. Genome sequence analysis of *P. indica* has revealed its potential as plant probiotic agent (Qiang et al., 2012). Complete genomes of many of the bacterial as well as fungal endophytes have been sequenced (**Table 1**), and the list is getting further populated. Available endophyte genomes serve as the model systems to study plant–microbe and microbe–microbe interactions. Further, individual genome sequences improve the data analysis in metaomics (metagenomics, transcriptomics, and proteomics) studies of plant associated microbes.

**Table 1:** List of endophytes whose genomes have been sequenced.

	Fungal endophytes	Reference
1	*Epichloe festucae* E2368	Schardl et al., 2009, 2010
2	*Piriformospora indica*	Zuccaro et al., 2011
3	*Ascocoryne sarcoides*	Gianoulis et al., 2012
4	*Penicillium aurantiogriseum* NRRL 62431	Yang Y. et al., 2014
5	*Shiraia* sp. slf14	Yang H. et al., 2014
6	*Harpophora oryzae*	Xu et al., 2014
7	*Pestalotiopsis fici*	Wang et al., 2015
8	*Rhodotorula graminis* WP1	Firrincieli et al., 2015
9	*Phialocephala scopiformis* DAOMC 229536	Walker et al., 2016
10	*Microdochium bolleyi* J235TASD1	David et al., 2016
11	*Xylona heveae*	Gazis et al., 2016

	**Bacterial endophytes**	**Reference**

1	*Azoarcus* sp. strainBH72	Krause et al., 2006
2	*Klebsiella pneumoniae* 342	Fouts et al., 2008
3	*Gluconacetobacter diazotrophicus* Pal5	Bertalan et al., 2009
4	*Enterobacter* sp. strain638	Taghavi et al., 2010
5	*Stenotrophomonas maltophilia* R551-3	
6	*Pseudomonas putida* W619	
7	*Serratia proteamaculans* 568	
8	*Azospirillum* sp. B510	Kaneko et al., 2010
9	*Bacillus subtilis*	Deng et al., 2011
10	*Variovorax paradoxus*	Han et al., 2011
11	*Herbaspirillum seropedicae* strain SmR1	Pedrosa et al., 2011
12	*Burkholderia phytofirmans* strain PsJN	Weilharter et al., 2011
13	*Stenotrophomonas maltophilia* RR-10	Zhu et al., 2012
14	*Enterobacter* sp. strain SST3	Gan et al., 2012
15	*Burkholderia* sp. strain KJ006	Kwak et al., 2012
16	*Enterobacter cloacae* subsp. Cloacae strainENHKU01	Liu et al., 2012
17	*Enterobacter radicincitans* DSM16656(T)	Witzel et al., 2012
18	*Rhizobium* sp. strain IRBG74	Crook et al., 2013
19	*Enterobacter cloacae* P101	Humann et al., 2013
20	*Pseudomonas poae* RE^∗^1-1-14	Muller et al., 2013
21	*Herbaspirillum frisingnse* GSF30	Straub et al., 2013
22	*Bacillus pumilus*	Jeong et al., 2014
23	*Paenibacillus* sp.P22	Hanak et al., 2014
24	*Pantoea agglomerans*	Gan et al., 2014
25	*Staphylococcus haemolyticus*	
26	*Pseudomonas* sp. strain RIT288 & RIT357	
27	*Microbacterium oleivorans*	
28	*Micrococcus luteus* strain RIT304, RIT305 & RIT324w	
29	*Janthinobacterium lividum*	
30	*Stenotrophomonas* sp.	
31	*Delftia* sp.	
32	*Sphingomonas* sp.	
33	*Exiguobacterium* sp.	
34	*Klebsiella variicola* DX120E	Lin et al., 2015
35	*Pseudomonas fluorescens* PICF7	Martinez-Garcia et al., 2015
36	*Kosakonia oryzae* K0348	Meng et al., 2015
37	*Raoultella terrigena* R1Gly	Schicklberger et al., 2015
38	*Paenibacillus dauci*	Wu et al., 2015
39	*Pantoea ananatis* AMG521	Megias et al., 2016
40	*Bacillus thuringiensis* KB1	Jeong et al., 2016
41	*Staphylococcus epidermidis* strains SE2.9; 4.6; 4.7 and 4.8	Chaudhry and Prabhu, 2016


Host plant genome evolution is also affected by endophytic colonization (Guo et al., 2015) whereas Zgadzaj et al. (2015) reported that host genetic factors control establishment of both endophyte and the symbiont within root-nodules, therefore host genome studies are also important for a clearer view.

## Multigenome Analysis

Comparative multigenome analysis is quite helpful in understanding genetic and metabolic diversity of similar or related microbes involved in different types of interactions with the plants as well as with animals. It seems that a fine line divides the colonization of host plant by a microbe as symptomless endophyte or a pathogen. Extensive comparative analysis of the genome of *Piriformospora indica* with that of fungi belonging to different classes, has revealed the presence of genes related to both saprotropism as well as biotropism lifestyle (Zuccaro et al., 2011). Interestingly, the microbe is equipped with the essentials for both the life styles, so there must be some external factors that make the microbe to choose one of them. Comparison of genomes of closely related species inhabiting different microbiomes is quite useful in disclosing the molecular determinants responsible for this distinction. Comparative genomics studies have revealed that difference in metabolic, secretory, transport, and surface attachment proteins are mainly responsible for selection of contrasting habitats. Monteiro et al. (2012) reported lipopolysaccharide and adhesins as potential molecular factors responsible for the contrasting phenotypic behavior of closely related species during host plant colonization as symbiont endophyte or as phytopathogen. Variation was observed in the distribution of essential genes related to signaling, surface attachment, secretion, and transport between the two strains of *Klebsiella pneumoniae* which revealed the divergences for the preferred lifestyle as plant endophyte for Kp342 and as a human pathogen for other strain MGH78578 (Fouts et al., 2008). These data suggest that Kp342 is well adapted to escape plant defense reactions and successfully establishes itself inside a plant. Monteiro et al. (2012) have identified the genes accounting for differences in the colonization patterns of two closely related species, i.e., endophytic *Herbaspirillum seropedicae* Smr1 and the phytopathogenic *H. rubrisubalbicans* M1, using suppression subtractive hybridisation (SSH). SSH is an effective technique for the analysis of genetic diversity among the microbes (Winstanley, 2002; Galbraith et al., 2004). SSH libraries are constructed to identify the DNA fragments present in one species and absent in other.

Genome comparison of endophytic isolates with their non-endophytic counterparts reveal features likely to be inevitable for establishing and further maintaining plant microbial interactions. Further, the study is also helpful to realize the genetic drivers of niche adaptation (Lopez-Fernandez et al., 2015). Genomes of closely related endophytic species, but exhibiting different functional roles in host plant can also be compared for determining the adaptability and evolution strategies. Tezerji et al. (2015) have compared the genomes of three strains of *P. ananatis.* The three strains were isolated as maize seed endophytes but exhibited different interaction strategies with the host plant. Genome comparisons revealed differences among the strains for secretory protein, integrase, transposase and phage related genes. Multigenome comparative analysis of more than ten members of Clavicipitaceae family, for gene clusters of four classes of alkaloids, revealed that variations in peripheral genes of the alkaloid loci are responsible for their pharmacological specificities (Schardl et al., 2013). Thus, comparative genome analysis of endophytes for different metabolite gene clusters can prove to be useful in understanding metabolic diversity among the members of a microbiome and thus, this knowledge can be exploited in metabolic engineering.

Pan genome studies have also opened a new window to closely observe genetic determinants of endophytism (Medlini et al., 2005; Mayer et al., 2014). Pan genome can be defined as an overall gene repertoire of a species which comprises of a core genome and an accessory genome. Core genome involves the genes present in all strains of the species whereas accessory genome involves genes unique to particular strains. Pan genome studies may therefore lead to the identification of signature genes responsible for adaptation and evolution of a microbe as an endophyte. Genome sequence studies are based on cultivation dependent approach and therefore, non-culturable endophytic microbial taxa remain untouched.

## Metagenomics

Metagenomics involves analysis of sequence information from microbial members of various ecological communities. It evades the need for isolation and cultivation of individual species. In order to understand and manipulate the contribution of endophytes to the host plant, it is important to uncover their metabolic potential and beneficial characteristics. However, determination of endophytic microbial functions is impeded by the non-culturable feature of many endophytes (Dinsdale et al., 2008). A metagenomics approach is quite helpful in unraveling the potential of uncultured microbial communities (endophytes) (Dinsdale et al., 2008), thus revealing the information beyond the genomic information of individual taxa. In this approach, DNA is extracted from the whole population and analyzed for its gene content. Sessitsch et al. (2012) unraveled the putative functional characteristics of the root endophytes of rice based on metagenome analysis. They reported numerous metabolic adaptations of endophytes to their microhabitat, thereby, suggesting high potential of the endophyte community in terms of plant-growth promotion, enhancement of plant stress resistance, bio control against pathogens and bioremediation.

Functional diversity has been maintained among microbial communities inhabiting different environments. Comparative metagenomics approach can be successfully used to study functional diversity among endophytes of same or the different host plants. Dinsdale et al. (2008) used the comparative metagenomics approach to describe the variations in functional potential of nine different microbiomes.

High throughput sequencing called next generation sequencing (NGS) has made metagenomic studies comparatively easier and catalyzed the rapid, unprecedented characterisation studies of microbiomes (Akinsanya et al., 2015). It has equipped researchers with a wide ranging tool for quick and affordable study of DNA sequences from an environmental sample (Jones, 2010). Four hundred and fifty-four sequencing has provided a convenient means for the characterisation of fungal communities (Jumpponen et al., 2010). Toju et al. (2013) described the community composition of root-associated fungi in a temperate forest in Japan. They demonstrated the coexistence of mycorrhizal fungi and endophytic fungi in roots of different plant species using 454 pyrosequencing techniques. Coexistence would surely involve complex interactions between the two ecotypes which can be further studied using metaproteomics, metatranscriptomics, or metaproteogenomics approach. One should be aware of the limitations regarding NGS technologies prior to using the same for experimental studies (Daniel et al., 2008; Jones, 2010). High ratio of sequences with no homolog in public databases is one of the major limitations of metagenomic studies. Genome sequencing studies of the strains collected from the same niche would overcome the limitation to an extent.

## Transcriptomics and Metatranscriptomics

Transcriptomics has been found as a feasible approach to study the microbial communities associated with different plants (Molina et al., 2012; Sheibani-Tezerji et al., 2015). It involves the comparative analysis of transcriptomes of groups of interacting species and helps to understand the response of microbial communities toward changing environments. While genome and metagenome based studies enumerate the presence or absence of specific genes, expression studies of specific genes in different microenvironments are essential to understand the endophytic phenomenon. Deep analysis of the differentially expressed genes in the host plant as well as symbiotic microbes would provide insight into the basic nature and mechanism of mutualistic relationships between the two. Dual RNA-seq transcriptional profiling gives better idea of gene expression in both the partners of symbiosis at a time. Camilios-Neto et al. (2014) have used dual RNA-seq technology for transcriptional profiling of wheat roots colonized by *Azospirillum brasilense* and observed upregulation of nutrient acquisition and cell cycle genes. RNA seq allows detection of more differentially expressed genes than microarray alone. Despite of more advantages of RNA seq, microarray is still more commonly used tool for transcriptional profiling because of the high cost and relatively difficult data storage and analysis in RNA seq technology. Metatranscriptomic analysis of soybean plant has revealed the presence of a number of small RNA sequences unrelated to soybean genome. Interestingly comparative analysis of the obtained sequences established the presence of various pathogenic, symbiotic and free living microbes in different samples of soybean plant (Molina et al., 2012).

Comparative transcriptome analysis of endophyte free and endophyte infected plants direct us toward understanding the basis of endophyte mediated disease resistance and plant growth promotion properties. Comparative studies regarding differential expression profiles of endophytes within and outside host plant can be helpful to identify interaction factors involved in maintaining the relationship. Conversely differential expression of different host plant genes in presence and absence of endophytes can also be studied. SSH, microarray analysis and SOLiD-SAGE like techniques can be successfully used for differential expression analysis (Johnson et al., 2004; Dinkins et al., 2010; Ambrose and Belanger, 2012). SOLiD-SAGE transcriptome analysis of endophyte free and *Epichloe festucae* infected *Festuca rubra* has revealed about two hundred plant associated genes that expressed differentially between the two plant samples (Ambrose and Belanger, 2012). Genome based studies make an important base for successful transcriptomics. Thus, combined genome and transcriptome analysis is more helpful in decoding the endophytic life style of symbionts.

## Proteomics and Metaproteomics

With the advancement in technology, post genomic analyses of the microbial communities are becoming popular as the genomics based analyses are unable to uncover the actual function of microbial communities *in situ*. Proteomics is defined as the large scale study of different proteins expressed by an organism (Wilkins et al., 1995) whereas metaproteomics involves identification of the functional expression of the metagenome and elucidation of the metabolic activities occurring within a community at the moment of sampling. It is also known as whole community proteomics. Maron et al. (2007) have stressed on the relevance of metaproteome analysis in identification of new functional, stress related genes and in relating genomic diversity with the functionality of the microbes inhabiting complex environments. Mass spectrometry (MS) has emerged as the unchallenged leader in the field, becoming the dominant technological platform for almost all proteomic measurements. Metaproteomics exploits the power of high performance MS for extensive characterization of the complete suite of proteins expressed by a microbial community in an environmental sample. Total proteins can be extracted from microenvironment either by direct or indirect lysis (Maron et al., 2007). Using direct lysis strategy total protein content can directly be extracted from plant endosphere under different natural and stress conditions and the protein fingerprint so obtained can be analyzed to study the impact on metabolite production potential of endophytes. Conversely, using indirect lysis method, total protein content can be extracted from preisolated endophytes under different stress conditions and comparison of the protein fingerprints obtained after 2,D-gel electrophoresis analysis, can be used to reveal the role of endophytes under different stress conditions (Bhuyan et al., 2015). Otherwise, total protein content of host plants in presence and absence of endophytes can also be assessed to identify actual specific proteins involved in interactions between the two. Lery et al. (2011) found 78 differentially expressed proteins between sugarcane-*Gluconacetobacter* interaction model and control cultures using Mass-spectrometry based proteomic analysis of the same. Proteome based studies are incomplete without genomic information. Moreover protein extraction and sample preparation is a difficult step in proteomic studies due to the presence of interfering substances like alkaloids, polyphenols, thick polysaccharides, lipids, organic acids, and other secondary metabolites. More information regarding the metagenomes of microbial communities from different environments is needed for the effectiveness of this technique in characterizing endophytic microbial communities.

## Metaproteogenomics

Metaproteogenomics links the proteome and the genome of the environmental samples and allows identification of more proteins (functions) than proteomics alone. It involves combinatorial study of metagenome and metaproteome of same sample. Knief et al. (2012) have used metaproteogenomic approach to study microbial communities in the phyllosphere and rhizosphere of rice. The results showed that despite the presence of nifH genes in both microenvironments, expression was found in rhizosphere only. If such an approach could be applied to study the endosphere, more significant data regarding the endophyte functionality can be collected. Characterization of the metaproteogenome is expected to provide data linking genetic and functional diversity of microbial communities. Proteins involved in plant endophyte interactions that could not be studied in cultivated isolates are new targets for functional studies. Plant associated bacterial protein secretion system can be successfully used for determining plant bacterial interactions (Downie, 2010). Delmotte et al. (2009) have successfully used community proteogenomics to identify the unique traits of phyllosphere bacteria. Bacterial proteogenomic pipeline and other tools are available for proteogenomic analysis studies (Uszkoreit et al., 2014). The technique offers insights into possible strategies adopted for endophytic lifestyle. The combined metagenome and metaproteome analysis would allow one to overcome the limitations of protein identifications as in metaproteomic approach due to non-availability of closely related reference genomes.

## Microarray-Based Techniques

Microarray technique has equipped the modern genome based studies with the tools for genome specific gene expression studies, endophyte gene profiling, exploration of host plant–symbiont interactions and many others for transcriptome analysis (Felitti et al., 2006). Barnett et al. (2004) used the dual genome Symbiosis Chip based tool to study symbiotic interactions. Symbiosis chip based studies allow simultaneous analysis of gene expression in both partners of the association and can easily be used to study the endophyte host interactions. Barnett et al. (2004) studied the coordinate differentiation and response generated from signal exchange between the two symbiotic partners simultaneously viz. α-proteobacterium *Sinorhizobium meliloti* and its legume partner *Medicago truncatula* during nodule development. They designed a custom Affymetrix Gene Chip with the complete *S. meliloti* genome and ≈10,000 probe sets for *M. truncatula*.

Genomic interspecies microarray hybridisation technique has proved to be useful in the characterisation of previously untouched genomes, provided that the genome of a close relative has already been fully sequenced (Dong et al., 2001). Microarray technique allows the identification of a number of genes in an uncharacterised genome without the need for genome sequencing. This technique finds more applications with sequencing of endophytic genomes (**Table 1**). Genes have been discovered efficiently in maize endophyte *K. pneumonia* 342 by hybridizing the DNA from KP342 to a microarray containing 96% of the annotated ORFs from *Escherichia coli* K12 (Dong et al., 2001). Microarray studies can be used to study the transcriptional changes induced by entry of endophytes in plants. These studies provide a new insight into the biology of endophyte host interaction and represent a step forward toward identification of host genes required for successful endophyte infestation. Felitti et al. (2006) described the potential of *Epichloe* and *Neotyphodium* endophyte cDNA microarrays (Nchip^TM^ and Endochip^TM^ microarrays) for genome wide transcriptome analysis. Microarray analysis of transcriptome of endophytic-*Pseudomonas* infected *Arabidopsis* revealed the upregulation of phytohormone production and nodule formation genes whereas ethylene responsive genes were found to be downregulated (Wang et al., 2005). Reference selection is a critical step in microarray studies as non-specific references may generate ambiguous results. However, non-availability or limited access to the specific gene expression/profiling databases have restricted such studies.

System biology science embraces four key technologies viz. genomics, transcriptomics, proteomics, and metabolomics. All the approaches along with their “meta-omic” partners (including metagenomics, metatranscriptomics, etc.) are in a state of expeditious expansion. While the individual type of data are useful, they are even more valuable when used in combination. Genomic information introduces just to the potential wealth hidden in a microenvironment in the form of molecular machinery but the actual expression and function remain unknown. On the other hand, transcriptome studies reveal genome expression under different environmental conditions without considering protein level regulation like post translational modifications, protein turnover, etc., Significantly, proteomic studies disclose functional gene products exploited by microbes for life processes. However, proteomic studies are consummated only after collating measured protein data (derived from proteomic studies) with predicted protein data (derived from genomic studies) (Hettich et al., 2013). However, transcriptomic and proteomic studies are ineffective without genome based studies. Moreover, complementing metagenomics data with the metatranscriptomic and metaproteomic data would generate complete view of the activities and potential of the endophytes. All techniques are interdependent and the data generated from one complement the another. Thus, combined analysis of data generated from different modern “omics” tools would prove to be helpful in solving the riddle of endophytism.

## Conclusion

Deep understanding of endophyte host interactions is the need of the hour in order to realize the use of endophytes as plant probiotics. By using a multidisciplinary approach, factors inevitable for both the establishment as well as maintenance of symbiotic association between the two can be better understood. Such studies are also important to elucidate that how the endophytes confer stress tolerance and growth promotion to its host plant. The complementary information generated from modern “omics” studies (discussed above) in association with other system biology techniques are inevitable to build up models to predict and explain endophyte mediated processes. This will also prove to be quite useful in revealing and better understanding of the network of the complex interactions of endophytes with the host plant and also other associated microbes. Plant–pathogen interaction studies can be used as a base model to understand plant–endophyte relationship. Advanced techniques can be used with the same accuracy for bacterial as well as fungal endophytes to reveal the genetic and metabolic potential, as well as ecology and evolution of endophytes. This can make us understand the role of such diverse microbial communities in the plant microbiome as well as in natural ecosystem, so that their biotechnological potential can be harnessed more efficiently and sustainably.

## Author Contributions

All authors listed, have made substantial, direct and intellectual contribution to the work, and approved it for publication.

## Conflict of Interest Statement

The authors declare that the research was conducted in the absence of any commercial or financial relationships that could be construed as a potential conflict of interest.

## Footnotes

1 http://mycor.nancy.inra.fr/blogGenomes/?page_id=149
